# Proper adjuvant therapy in patients with borderline resectable and locally advanced pancreatic cancer who had received neoadjuvant FOLFIRINOX

**DOI:** 10.3389/fonc.2022.945829

**Published:** 2022-09-20

**Authors:** Jin Ho Choi, Min Kyu Kim, Sang Hyub Lee, Jin Woo Park, Namyoung Park, In Rae Cho, Ji Kon Ryu, Yong-Tae Kim, Jin-Young Jang, Wooil Kwon, Hongbeom Kim, Woo Hyun Paik

**Affiliations:** ^1^ Department of Internal Medicine and Liver Research Institute, Seoul National University Hospital, Seoul National University College of Medicine, Seoul, South Korea; ^2^ The Armed Forces Medical Command, Ministry of National Defense, Gyeonggi-do, South Korea; ^3^ Department of Pathology, Seoul National University Hospital, Seoul National University College of Medicine, Seoul, South Korea; ^4^ Department of Surgery, Seoul National University Hospital, Seoul National University College of Medicine, Seoul, South Korea

**Keywords:** pancreatic cancer, neoadjuvant therapy, FOLFIRINOX, adjuvant chemo- therapy, locally advanced pancreatic cancer (LAPC), borderline resectable pancreatic adenocarcinoma

## Abstract

**Background:**

The complete resection rate of pancreatic cancer has increased because of the advent of efficacious first-line treatments for unresectable pancreatic cancer. Still, strategies regarding adjuvant therapy after neoadjuvant FOLFIRINOX treatment remain to be established.

**Methods:**

Data on 144 patients with borderline resectable and locally advanced pancreatic cancer who underwent resection after neoadjuvant FOLFIRINOX between January 2013 and April 2021 were retrospectively reviewed.

**Results:**

Among the study patients, 113 patients (78.5%) were diagnosed with borderline resectable pancreatic cancer and 31 patients (21.5%) were diagnosed with locally advanced pancreatic cancer. Seventy-five patients (52.1%) received radiotherapy before surgery. After radical resection, 84 patients (58.3%) received 5-fluorouracil-based adjuvant therapy and 60 patients (41.7%) received non-5-fluorouracil-based adjuvant therapy. Adjuvant therapy with 5-fluorouracil-based regimen [hazard ratio (HR), 0.43 (95% CI, 0.21–0.87); p = 0.019], preoperative assessment as locally advanced pancreatic cancer [HR, 2.87 (95% CI, 1.08–7.64); p = 0.035], positive resection margin [HR, 3.91 (95% CI, 1.71–8.94); p = 0.001], and presence of pathologic lymph node involvement [HR, 2.31 (95% CI, 1.00–5.33), p = 0.050] were associated with decreased recurrence-free survival. Adjuvant therapy with 5-fluorouracil-based regimen [HR, 0.35 (95% CI, 0.15–0.84); p = 0.018], positive resection margin [HR, 4.14 (95% CI, 1.75–9.78); p = 0.001], presence of pathologic lymph node involvement [HR, 3.36 (95% CI, 1.23–9.15); p = 0.018], poor differentiation [HR, 5.69 (95% CI, 1.76–18.36); p = 0.004], and dose reduction during adjuvant therapy [HR, 1.78 (95% CI, 1.24–24.37); p = 0.025] were associated with decreased overall survival.

**Conclusions:**

The 5-fluorouracil-based adjuvant therapy seems to be the proper adjuvant therapy for patients who received neoadjuvant FOLFIRINOX for borderline resectable and locally advanced pancreatic cancer.

## Introduction

Pancreatic cancer (PC) is the fourth leading cause of death from cancer in the United States (1). Surgical resection is the only potential curative method; however, resectable PC accounts for only approximately 10%–15% of total PC (2). Furthermore, the prognosis of patients who have undergone surgery is extremely poor, with a 5-year survival rate of 10% and a recurrence rate of 80% (1, 3).

Neoadjuvant therapy (NAT) rather than upfront surgery is recommended for treating non-metastatic PC with improved overall survival (OS) and increased tumor-free resection margin (RM) after surgery (4–6). Among two preferred NATs, FOLFIRINOX and gemcitabine plus nab-paclitaxel (6), robust clinical trial data comparing these two regimens as NATs are still lacking, although FOLFIRINOX has shown its efficacy through several studies (7, 8). The use of various regimens of adjuvant therapy (AT) following surgical resection of PC has improved OS and disease-free survival (9–12); however, data were gathered from patients who underwent upfront resection rather than NAT and subsequent resection. Until now, there are no prospective data regarding optimal AT for patients undergoing surgical resection after NAT. Furthermore, data on predictive factors for tumor recurrence and OS in these patients are scarce despite their clinical importance.

This study aimed to assess the prognostic factors and identify the proper AT for those undergoing neoadjuvant FOLFIRINOX and subsequent resection.

## Materials and methods

### Patient and study design

Patients diagnosed with borderline resectable (BR) or locally advanced (LA) PC and receiving neoadjuvant FOLFIRINOX at Seoul National University Hospital from January 2013 until December 2020 were included in this study. The exclusion criteria were as follows: 1) patients who failed to receive curative resection, 2) patients who had a history of other malignancy within the five most recent years, 3) patients diagnosed with metastatic PC, 4) patients lost to follow-up, 5) patients who were followed up less than 2 months after surgery, and 6) patients who had a history of pancreatic surgery for other diseases. This study protocol was approved by the institutional review board of Seoul National University Hospital (IRB No. 1711-107-901). Data of the study patients were retrospectively collected from electronic medical records. Age, Eastern Cooperative Oncology Group performance status, tumor size, lymph node (LN) status, response to NAT, initial/perioperative serum carbohydrate antigen 19-9 (CA 19-9) levels, and pathologic reports on surgical specimens were collected, and the data were analyzed.

### Assessment and definition

The patient’s response to anticancer therapy was evaluated according to the RECIST 1.1 criteria (13). Recurrence-free survival (RFS) was defined as the duration in months from the date of surgery to the date of recurrence or the date of the last follow-up. OS was defined from the date of diagnosis to the date of death in months or the date of last the follow-up. Survival data were gathered from the national database from the Ministry of Public Administration. The response to NAT was evaluated by the College of American Pathologists (CAP) score (14). In the CAP scoring system, the assessment of tumor response was performed through a four-step system: no viable cancer cells (grade 0), single cells or rare small groups of cancer cells (grade 1), residual cancer with evident tumor regression but more than single cells or rare small groups of cancer cells (grade 2), and extensive residual cancer with no evident tumor regression (grade 3). There were various scoring systems of tumor response after NAT in resected PC, and according to a recent systematic review, the CAP scoring system showed the lowest risk of bias with good applicability system among existing scoring systems (15). Postoperative TNM stage was defined using the American Joint Committee on Cancer Eighth guidelines (16). Assessment of the adverse events followed the National Cancer Institute Common Toxicity Criteria (version 5.0) (17).

### Treatment for borderline resectable or locally advanced pancreatic cancer

Study patients were treated with the neoadjuvant FOLFIRINOX regimen that was used in the previous study (18). Each patient underwent surgery of PC after neoadjuvant FOLFIRINOX based on the decision of a multidisciplinary team. Tumor resectability was defined following the National Comprehensive Cancer Network criteria (19). BRPC and LAPC were defined on the basis of abutment or involvement of adjacent major vessels (19). Surgical extent was evaluated by consensus of the International Study Group for Pancreatic Surgery (20).

### Statistical analysis

Continuous variables were provided as median values with a 95% confidence interval (95% CI), and categorical variables were provided as numbers and proportions (%). Kaplan–Meier survival analysis and log-rank test were used to compare RFS and OS between groups. Further grouped survival analysis according to pathologic response (CAP score) and AT regimen was also performed. Multivariable Cox proportional hazards analysis was conducted using variables considered to be clinically meaningful factors affecting RFS and OS. Relative hazard ratios (HRs) of recurrence and survival were analyzed with various clinical factors. Multivariable analyses predicting recurrence and survival were also conducted with statistically significant in univariable analysis or factors thought to affect clinical outcomes. Subgroup analysis for patients who underwent neoadjuvant radiotherapy in R0 resection rate, risk for recurrence or death, and pattern of tumor recurrence was conducted. Also, subgroup analysis of prognostic factors for recurrence and death by multivariable Cox proportional hazards analysis except of patients who underwent AT without FOLFIRINOX among those in the 5-fluorouracil (5-FU)-based AT group was conducted. A p value <0.05 was considered to indicate statistical significance. All statistical analyses were conducted using SPSS v.23.0 (IBM Corp., Armonk, NY, USA).

## Results

### Study patients and baseline characteristics

**Figure 1:**
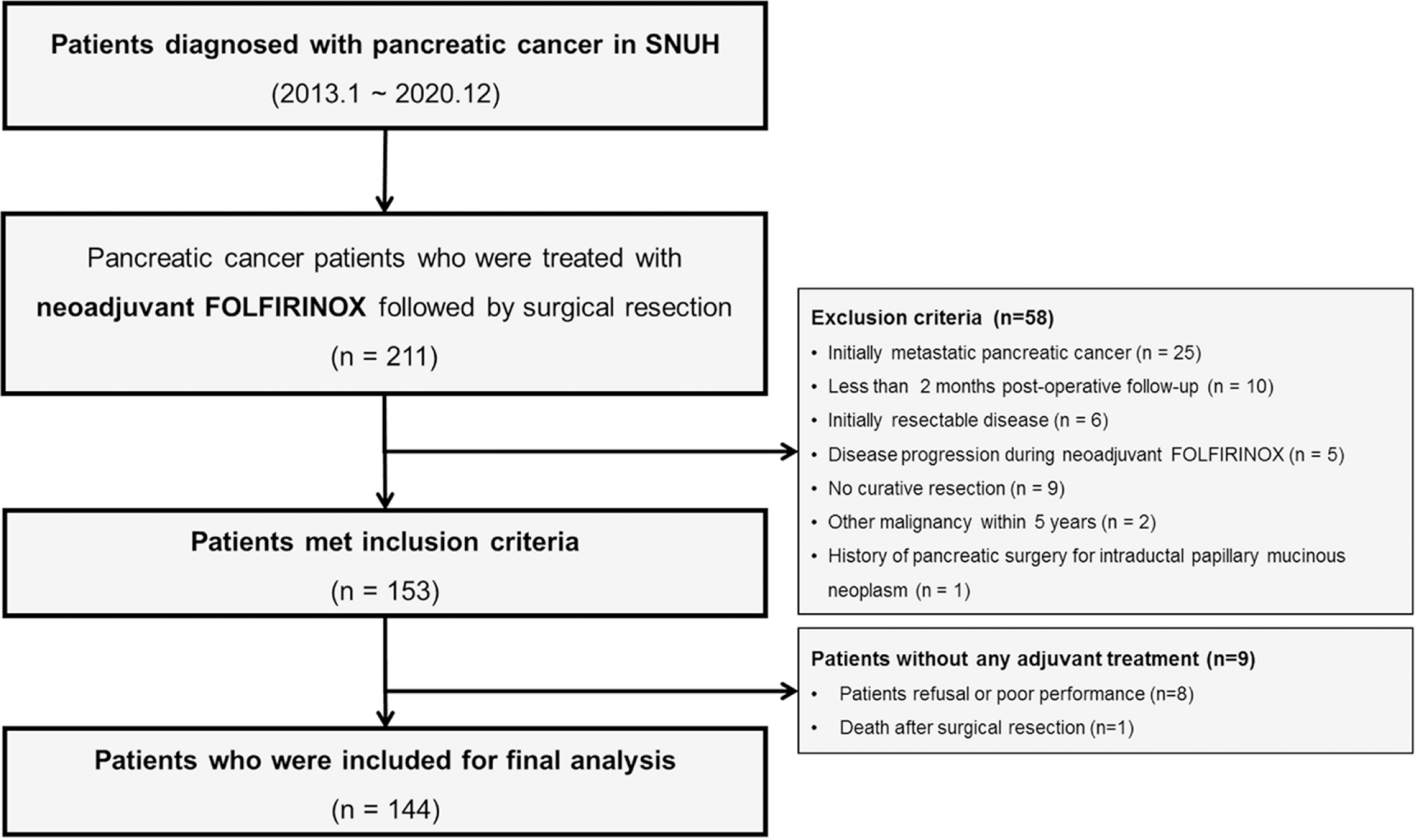
Flowchart of this study.

A total of 211 patients who were diagnosed with PC were treated with FOLFIRINOX followed by surgery during the study period, and 144 patients were finally included for this study (**Figure 1**). **Table 1** summarizes the baseline characteristics of the study patients. There were 74 male patients (51.4%), and the median age was 64 years. One hundred forty-two patients (98.6%) showed good performance status (ECOG <2) before neoadjuvant chemotherapy. One hundred thirteen patients (78.5%) were diagnosed with BRPC, and 31 patients (21.5%) were diagnosed with LAPC. The initial CA 19-9 level at diagnosis was 349.0 U/mL [interquartile range (IQR) 25.75–1,763.5 U/mL]. The patients underwent radical resection after a median of eight cycles (range, 3–30) of neoadjuvant FOLFIRINOX therapy, and 40 patients (27.8%) received the reduced dose at least once for NAT. Twenty-three patients (16.0%) experienced severe adverse events more than grade 2 during neoadjuvant chemotherapy. Seventy-five patients (52.1%) received radiotherapy before surgery. One patient (0.7%) showed a complete response, and 42 patients (29.2%) showed a partial response after NAT, whereas 101 patients (70.1%) remained having stable disease. Prior to surgical resection, 120 patients (83.3%) were reevaluated with BRPC and 24 patients (16.7%) with LAPC. The median time from the date of diagnosis to the date of resection was 6.2 months (range, 2.83–21.73). After radical resection, 84 patients (58.3%) received 5-FU-based AT and 60 patients (41.7%) received non-5-FU-based AT. In the 5-FU-based AT group, a total of 51 patients (60.7%) were treated with FOLFIRINOX, 17 patients (20.2%) with 5-FU with leucovorin, 14 patients (16.7%) with concomitant chemoradiotherapy using 5-FU or capecitabine, and two patients (2.4%) with tegafur/gimeracil/oteracil potassium. In the non-5-FU-based AT group, a total of 49 patients (81.7%) were treated with gemcitabine, 10 patients (16.7%) with concomitant chemoradiotherapy using gemcitabine, and one patient (1.7%) with gemcitabine and nab-paclitaxel.

**Table 1:** Baseline characteristics and treatment information.

Total N = 144			N (%)
Baseline Characteristics			
**Sex**	Men		74 (51.4)
**Age (median), years**			64
**Performance before neoadjuvant chemotherapy**	ECOG <2		142 (98.6)
	ECOG ≥2		2 (1.4)
**Tumor site**	Head and uncinate process		104 (72.2)
	Body and tail		40 (27.8)
**Vessel involvement at diagnosis**	Major artery		28 (19.4)
	Major vein		72 (50.0)
	Both		44 (30.6)
**Initial resectability**	BRPC		113 (78.5)
	LAPC		31 (21.5)
**CA 19-9 (median), U/mL**	result at diagnosis		349.0 (IQR 25.75–1,763.5)
**Treatment information**	
**Neoadjuvant FOLFIRINOX**	Cycle, median (range)		8 (3–30)
	Dose reduction		40 (27.8)
**Severe adverse events during neoadjuvant FOLFIRINOX**	≥ Grade 3		23 (16.0)
**Neoadjuvant radiotherapy**			75 (52.1)
**Time to surgical resection after diagnosis (median), months**			6.15 (2.83–21.73)
**Response assessment by RECIST for neoadjuvant treatment**	CR		1 (0.7)
	PR		42 (29.2)
	SD		101 (70.1)
**Preoperative resectability**	BRPC		120 (83.3)
	LAPC		24 (16.7)
**Surgical resection**	Pancreaticoduodenectomy		36 (25.0)
	PPPD		60 (41.7)
	Distal pancreatectomy		34 (23.6)
	RAMPs		3 (2.1)
	Subotal pancreatectomy		4 (2.8)
	Total pancreatectomy		7 (4.9)
**Adjuvant treatment regimen**	5-FU-based		84 (58.3)
	FOLFIRINOX		51 (60.7)
	5-FU + leucovorin		17 (20.2)
	CCRT with 5-FU or capecitabine		14 (16.7)
	Tegafur/gimeracil/oteracil potassium		2 (2.4)
	Non-5-FU-based		60 (41.7)
	Gemcitabine		49 (81.7)
	CCRT with gemcitabine		10 (16.7)
	Gemcitabine + nab-Paclitaxel		1 (1.7)
	Dose reduction		12 (8.3)
**Performance before adjuvant treatment**	ECOG <2		128 (88.9)
	ECOG ≥2		16 (11.1)
**Time to adjuvant treatment after surgery (median), days**	5-FU-based		45 (IQR 38–59)
	Non-5-FU-based		42 (IQR 35–53.5)

BR, borderline resectable; CR, complete response; CCRT, concurrent chemoradiotherapy; ECOG, Eastern Cooperative Oncology Group; FU, fluorouracil; LA, locally advanced; PC, pancreatic cancer; PPPD, pylorus-preserving panceraticoduodenectomy; PR, partial response; RAMPs, radical antegrade modular pancreatosplenectomy; RT, radiotherapy; SD, stable disease; IQR, interquartile range; RECISIT, response evaluation criteria in solid tumor.

During a median of 23.9 months (range, 9.1–91.0) of follow-up period, 47 patients (32.6%) died and 58 patients (40.3%) experienced recurrence of the cancer. Sixty patients (41.7%) underwent extended resection. Negative RM was confirmed in 125 (86.8%) patients. The distribution of pathologic T stage was as follows: four (2.8%) patients were T0 (no tumor), 56 (38.9%) patients were T1, 65 (45.1%) patients were T2, 15 (10.4%) patients were T3, and four (2.8%) patients were T4. Also, the report of pathologic N stage data revealed 91 (63.2%) patients with N0, 48 (33.3%) with N1, and five (3.5%) with N2. The CAP scores of the surgical specimens were as follows: four (2.8%) patients had a score of 0, 41 (28.5%) patients had a score of 1, 54 (37.5%) patients had a score of 2, and 45 (31.3%) patients had a score of 3. Well-differentiated cancer was found in 14 (9.7%) patients, moderately differentiated cancer in 110 (76.4%) patients, and poorly differentiated cancer in 16 (11.1%) patients (**Table 2**).

**Table 2:** Results of surgical pathology and clinical outcomes.

		Total N = 144, N (%)
**Death**		47 (32.6)
**Recurrence**	Total	58 (40.3)
	Regional recurrence	19 (32.8)
	Distant metastasis	35 (60.3)
	Both	4 (6.9)
**Surgery to recurrence period**	Median (range)	12.9 (2.1–63.0) months
**Follow-up period**	Median (range)	23.9 (9.1–91.0) months
**Resection extent**	Extended resection	60 (41.7)
	Standard resection	84 (58.3)
**Resection margin**	R0	125 (86.8)
	R1	19 (13.2)
**Stage ypT**	T0 (no tumor)	4 (2.8)
	T1	56 (38.9)
	T2	65 (45.1)
	T3	15 (10.4)
	T4	4 (2.8)
**Stage ypN**	N0	91 (63.2)
	N1	48 (33.3)
	N2	5 (3.5)
**CAP score**	0	4 (2.8)
	1	41 (28.5)
	2	54 (37.5)
	3	45 (31.3)
**Differentiation**	WD	14 (9.7)
	MD	110 (76.4)
	PD	16 (11.1)
	No tumor	4 (2.8)
**Histologic type**	Ductal adenocarcinoma	132 (91.7)
	IPMN associated invasive carcinoma	3 (2.1)
	Adenosquamous carcinoma	2 (1.4)
	Acinar cell carcinoma	1 (0.7)
	Mucinous carcinoma	1 (0.7)
	Mixed adenocarcinoma and NEC	1 (0.7)

CAP, College of American Pathologists; IPMN, intraductal papillary mucinous neoplasm; MD, moderately differentiated; NEC, neuroendocrine carcinoma; PD, poorly differentiated; WD, well differentiated.

### Result of survival outcomes

**Figure 2:**
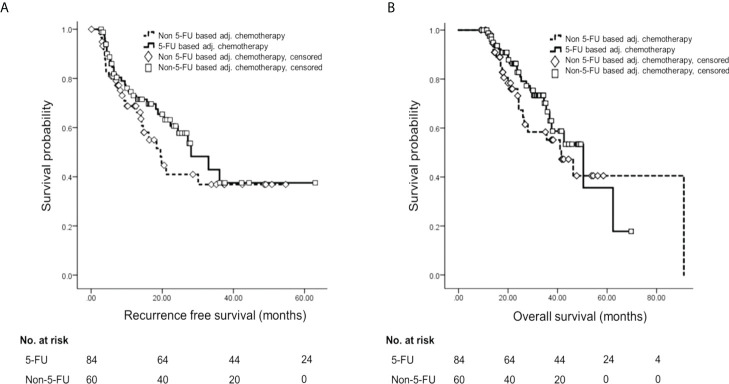
Survival analysis by Kaplan–Meier method and multivariable Cox proportional hazards analysis according to adjuvant treatment. **(A)** Kaplan–Meier curve of RFS according to adjuvant chemotherapy regimen. **(B)** Kaplan–Meier curve of OS according to adjuvant chemotherapy regimen. 5-FU, 5-fluorouracil; RFS, recurrence-free survival; OS, overall survival.

Among patients who experienced recurrence, regional recurrence occurred in 19 (32.8%) patients, distant metastasis in 35 (60.3%) patients, and both in four (6.9%) patients. RFS did not show a statistically significant difference according to AT (for 5-FU-based, median 28.1 ± 4.85 months, 95% CI 18.60–37.60; for non-5-FU-based, median 19.5 ± 3.13 months, 95% CI 13.40–25.60; p = 0.240) **(** **Figure 2A** **).** Also, OS did not show a statistically significant difference according to AT regimen (for 5-FU-based, median 50.4 ± 8.68 months, 95% CI 33.38–67.42; for non-5-FU-based, median 41.6 ± 6.31 months, 95% CI 29.13–53.87; p = 0.282) **(** **Figure 2B** **).**

**Figure 3:**
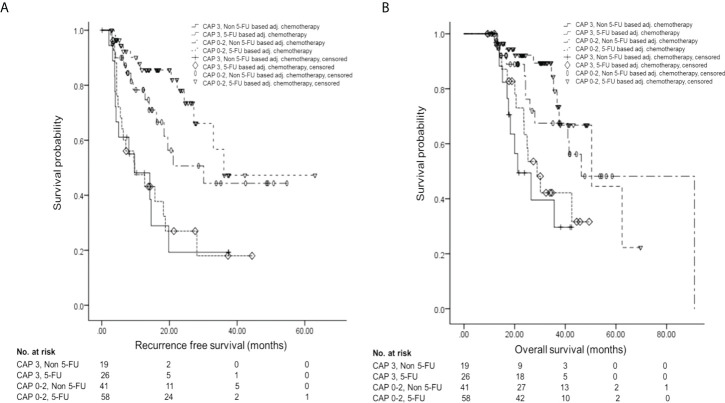
Survival analysis by the four groups according to pathologic response and AT regimen. **(A)** Kaplan–Meier curve of RFS according to groups. **(B)** Kaplan–Meier curve of OS according to groups. AT, adjuvant therapy; 5-FU, 5-fluorouracil; RFS, recurrence-free survival; OS, overall survival.

Further survival analyses were performed by the four groups according to pathologic response (CAP scores 0–2 vs. CAP score 3) and AT regimen (5-FU-based vs. non-5-FU-based). RFS showed a statistically significant difference among the groups [for CAP 3 and non-5-FU-based, median 9.7 ± 4.58 months, 95% CI 0.71–18.69; for CAP 3 and 5-FU-based, median 9.7 ± 4.37 months, 95% CI 1.14–18.26; for CAP 0–2 and non-5-FU-based, median 30.1 ± 9.59 months, 95% CI 11.30–48.90; for CAP 0–2 and 5-FU-based, median 36.1 months (standard deviation not applicable), 95% CI 27.2–not applicable; p < 0.001] **(** **Figure 3A** **).** Also, OS showed a statistically significant difference among the groups (for CAP 3 and non-5-FU-based, median 21.2 ± 4.92 months, 95% CI 11.56–30.84; for CAP 3 and 5-FU-based, median 28.9 ± 3.59 months, 95% CI 21.86–35.94; for CAP 0–2 and non-5-FU-based, median 46.3 ± 10. 34 months, 95% CI 26.04–66.56; for CAP 0–2 and 5-FU-based, median 50.4 ± 11.20 months, 95% CI 28.45–72.35; p = 0.001) **(** **Figure 3B** **).**

### Prognostic factors associated with recurrence-free survival and overall survival after resection

The results of relative HRs of RFS by univariable and multivariable analyses were shown in **Table 3**. In this multivariable model, we found that 5-FU-based AT [HR, 0.43 (95% CI, 0.21–0.87); p = 0.019], preoperative LAPC status [HR, 2.87 (95% CI, 1.08–7.64); p = 0.35], positive RM [HR, 3.91 (95% CI, 1.71–8.94); p = 0.001], and the presence of pathologic LN involvement [HR, 2.31 (95% CI, 1.00–5.33); p = 0.050] were associated with RFS.

**Table 3:** Prognostic factors for recurrence by multivariable Cox proportional hazards analysis.

Covariates	Univariable		Multivariable	
	HR (95% CI)	p-value	HR (95% CI)	p-value
**Age over 65 years (vs. 65 years and younger)**	0.86 (0.50–1.47)	0.572		
**Women (vs. men)**	0.86 (0.51–1.45)	0.577		
**Body and tail cancer (vs. head and uncinate)**	1.00 (0.56–1.79)	0.989		
**LA as initial resectability (vs. BR)**	0.98 (0.53–1.83)	0.961		
**Neoadjuvant RT (vs. No neoadjuvant RT)**	0.75 (0.44–1.28)	0.290		
**Neoadjuvant chemotherapy dose reduction (vs. standard dose)**	1.09 (0.63–1.89)	0.766	1.08 (0.51–2.26)	0.849
**LA as preoperative resectability (vs. resectable or BR)**	1.42 (0.75–2.68)	0.280	2.87(1.08–7.64)	0.035
**Objective response to neoadjuvant therapy (vs. stable disease)**	0.53 (0.27–1.05)	0.067	1.54 (0.64–3.70)	0.339
**Extended resection (vs. standard resection)**	1.12 (0.66–1.88)	0.680	1.17 (0.58–2.37)	0.655
**CAP score 0–2 (vs. 3)**	0.30 (0.18–0.50)	< 0.001	0.48 (0.22–1.05)	0.067
**Resection margin R1 (vs. R0)**	3.39 (1.84–6.24)	< 0.001	3.91 (1.71–8.94)	0.001
**Stage ypT2–4 (vs. ypT0 or ypT1)**	2.74 (1.50–5.01)	0.001	1.71 (0.82–3.54)	0.152
**Stage ypN1 or ypN2 (vs. ypN0)**	3.12 (1.85–5.26)	< 0.001	2.31 (1.00–5.33)	0.050
**Initial CA 19-9 ≥350 U/mL (vs. <350 U/mL)**	1.32 (0.78–2.22)	0.307		
**Postoperative CA19-9 normalization (vs. not)**	0.40 (0.22–0.74)	0.004	0.53 (0.27–1.06)	0.073
**Differentiation PD (vs. WD~MD)**	3.70 (1.95–7.03)	< 0.001	2.17 (0.81–5.82)	0.125
**5-FU-based adjuvant therapy (vs. non-5-FU-based)**	0.73 (0.44–1.23)	0.243	0.43 (0.21–0.87)	0.019
**Adjuvant therapy with FOLFIRINOX (vs. non-5-FU-based)**	0.59 (0.31–1.11)	0.103		
**Adjuvant chemotherapy dose reduction (vs. standard dose)**	0.81 (0.29–2.24)	0.684	1.99 (0.41–9.60)	0.390
**Performance before adjuvant therapy; ECOG 0 or 1 (vs. ECOG ≥2)**	1.13 (0.51–2.51)	0.760	2.98 (0.89–9.92)	0.076
**Adverse events with severity of grade 3 or more during neoadjuvant therapy (vs. grade 2 and less)**	0.62 (0.28–1.37)	0.236		

BR, borderline resectable; CAP, College of American Pathologists; ECOG, Eastern Cooperative Oncology Group; FU, fluorouracil; LA, locally advanced; MD, moderately differentiated; PD, poorly differentiated; RT, radiotherapy; WD, well differentiated; HR, hazard ratio; CI, confidence interval.

The results of relative HRs of OS by univariable and multivariable analyses were shown in **Table 4**. In this multivariable model, 5-FU-based AT [HR, 0.35 (95% CI, 0.15–0.84); p = 0.018], positive RM [HR, 4.14 (95% CI, 1.75–9.78); p = 0.001], presence of pathologic LN involvement [HR, 3.36 (95% CI, 1.23–9.15); p = 0.018], poor differentiation [HR, 5.69 (95% CI, 1.76–18.36); p = 0.004], and dose reduction during AT [HR, 1.78 (95% CI, 1.24–24.37); p = 0.025] were associated with OS.

**Table 4:** Prognostic factors for death by multivariable Cox proportional hazards analysis.

Covariates	Univariable		Multivariable	
	HR (95% CI)	p-value	HR (95% CI)	p-value
**Age over 65 years (vs. 65 years and younger)**	1.12 (0.61–2.04)	0.725		
**Women (vs. men)**	0.81 (0.45–1.46)	0.478		
**Body and tail cancer (vs. head and uncinate)**	0.71 (0.35–1.43)	0.335		
**LA as initial resectability (vs. BR)**	0.77 (0.38–1.56)	0.465		
**Neoadjuvant RT (vs. no neoadjuvant RT)**	1.27 (0.70–2.33)	0.435		
**Neoadjuvant chemotherapy dose reduction (vs. standard dose)**	1.21 (0.67–2.21)	0.527	0.71 (0.29–1.74)	0.711
**LA as preoperative resectability (vs. resectable or BR)**	1.14 (0.55–2.36)	0.734	1.64 (0.51–5.21)	0.404
**Objective response to neoadjuvant therapy (vs. stable disease)**	0.52 (0.22–1.23)	0.135	0.48 (0.16–1.45)	0.192
**Extended resection (vs. standard resection)**	1.43 (0.80–2.56)	0.225	2.29 (0.91–5.79)	0.080
**CAP score 0–2 (vs. 3)**	0.32 (0.18–0.58)	< 0.001	0.99 (0.38–2.58)	0.989
**Resection margin R1 (vs. R0)**	3.03 (1.63–5.63)	< 0.001	4.14 (1.75–9.78)	0.001
**Stage ypT2-4 (vs. ypT0-1)**	1.69 (0.91–3.14)	0.095	1.27 (0.56–2.90)	0.568
**Stage ypN1-2 (vs. ypN0)**	3.26 (1.80–5.91)	< 0.001	3.36 (1.23–9.15)	0.018
**Initial CA 19-9 ≥350 U/mL (vs. <350 U/mL)**	0.94 (0.52–1.68)	0.827		
**Postoperative CA19-9 normalization (vs. not)**	0.52 (0.26–1.04)	0.064	0.62 (0.28–1.39)	0.249
**Differentiation PD (vs. WD~MD)**	3.51 (1.81–6.80)	< 0.001	5.69 (1.76–18.36)	0.004
**5-FU-based adjuvant therapy (vs. non-5-FU-based)**	0.73 (0.41–1.30)	0.284	0.35 (0.15–0.84)	0.018
**Adjuvant therapy with FOLFIRINOX (vs. non-5-FU-based)**	0.75 (0.38–1.47)	0.400		
**Adjuvant chemotherapy dose reduction (vs. standard dose)**	1.65 (0.73–3.72)	0.230	5.49 (1.24–24.37)	0.025
**Performance before adjuvant therapy; ECOG 0 or 1 (vs. ECOG ≥2)**	0.96 (0.42–2.16)	0.911	1.78 (0.57–5.57)	0.324
**Adverse events with severity of grade 3 or more during neoadjuvant therapy (vs. grade 2 and less)**	0.50 (0.21–1.21)	0.123		

BR, borderline resectable; CAP, College of American Pathologists; ECOG, Eastern Cooperative Oncology Group; FU, fluorouracil; LA, locally advanced; MD, moderately differentiated; PD, poorly differentiated; RT, radiotherapy; WD, well differentiated; HR, hazard ratio; CI, confidence interval.

### Results of subgroup analyses

Subgroup analysis for patients who underwent neoadjuvant radiotherapy showed no differences in R0 resection rate, risk for recurrence or death, and pattern of tumor recurrence (**Supplementary Table S1**). Among the patients who underwent neoadjuvant radiotherapy, patients with 5-FU-based AT showed a lower risk of recurrence in comparison with patients with non-5-FU-based AT but no differences in other clinical outcomes.

Subgroup analyses of prognostic factors for recurrence and death by multivariable Cox proportional hazards analysis except of patients who underwent AT without FOLFIRINOX among those in the 5-FU-based AT group were conducted (**Supplementary Table S2**). AT with FOLFIRINOX and resection margin of R0 were associated with a lower risk of tumor recurrence, and AT with FOLFIRINOX, resection margin of R0, postoperative CA 19-9 normalization, and not poorly differentiated were associated with a lower risk of death.

## Discussion

Over the recent years, the number of unresectable PC patients undergoing NAT and subsequent resection is expanding because of advanced clinical outcomes of chemotherapy, and FOLFIRINOX is one of the most effective options in NAT (6, 7). Accordingly, proper AT for these patients is critically necessary. This study targeted this particular group of patients and concluded that AT with 5-FU is associated with a favorable survival according to the result from multivariable Cox proportional hazards analysis, along with negative RM, negative LN involvement for prolonged RFS and OS. Furthermore, preoperative assessment as BRPC was associated with prolonged RFS, and better differentiation and maintenance of standard-dose AT were associated with prolonged OS.

The recurrence rate and survival outcomes in this study were in line with those of previous studies (3, 21–27). Our study showed better OS than the reported OS of the up-front surgery strategy (5), supporting that neoadjuvant FOLFIRINOX treatment seems to be a better option than up-front surgery. The R0 resection rate reported in our study was also consistent with previous meta-analysis data (8). Therefore, it is essential to suggest the reasonable criteria to determine the proper strategy of adjuvant treatment for better outcomes in patients who underwent neoadjuvant chemotherapy. Through this study, we investigated the predictive factors by closely examining a wide variety of clinical and pathologic features.

Several factors including the absence of tumor-associated LN involvement, normalized CA 19-9 level after surgery, a negative RM, and pathologic response to NAT were proposed as prognostic factors after NAT in BRPC (26, 28, 29). We found that AT with 5-FU, clear RM, and no pathologic LN involvement were a favorable prognostic factor for RFS and OS in our study patients. One previous study reported that AT after neoadjuvant FOLFIRINOX therapy followed by surgery was effective for the subgroup of patients with LN involvement (27). However, there was no difference in the effect of the different ATs according to LN involvement in this study. Most of the patients in AT with 5-FU groups use FOLFIRINOX, and the excellence of this regimen was shown in the same context as demonstrated in the results of the PRODIGE 24/CCTG PA6 trial (12).

AT should be performed in a patient-stratified manner including performance status and patient tolerability, as it could be ineffective in the subgroup of patients with resected PC after neoadjuvant FOLFIRINOX. As recommended by the National Comprehensive Cancer Network guidelines (19), it still seems reasonable to determine adjuvant chemotherapy based on the response to neoadjuvant chemotherapy, and the results of this study are consistent with this recommendation. However, despite the promising result from the survival analysis by groups according to pathologic response and AT with 5-FU and univariable analysis, pathologic response (CAP score 0–2) and objective response for neoadjuvant FOLFIRNOX did not maintain the effectiveness of prognosis prediction in multivariable analysis.

Although most people using FOLFIRINOX were included, it may be crucial to include 5-FU for AT according to the result of this study. Recently, a basic research with 10 patient-derived PC organoids reported results in line with the overall observations in this study that the resistance to oxaliplatin and irinotecan was developed in organoids from patients who received neoadjuvant FOLFIRINOX, but 5-FU treatment responses were similar between organoids from naive and FOLFIRNOX-treated patients (30). Gemcitabine of better tolerability may be considered an alternative AT, especially for patients with poor performance, since FOLFIRINOX treatment is associated with more severe adverse events (12). Furthermore, completion of the planned AT was proven to be an independent prognostic factor for prolonged OS (31). Considering that a 5-FU-based AT with higher toxicity can result in dropping out midway through therapy, we anticipate that a gemcitabine-based AT for non-responders to neoadjuvant FOLFIRINOX might be a better option. Further research into subgroup selection for AT along with a structured algorithm is needed.

There were several limitations. First, the study was a retrospective study conducted in a single center. Since only the patients who underwent neoadjuvant FOLFIRINOX therapy were enrolled in this study, the sample size was small. Second, we only analyzed those who completed resection and received AT, which could introduce selection bias, and this resulted in a better survival. However, we adhered to this study design for the purpose of conducting research targeting patients in need of AT most in a real clinical setting. In addition, since the majority of the non-5-FU-based AT group did not respond to the neoadjuvant FOLFIRINOX, FOLFIRINOX was not selected as an AT after surgery, and the possibility that this could lead to selection bias should be considered. However, tumor response to neoadjuvant FOLFIRINOX was not a significant predictive factor in the multivariable analysis.

In conclusion, among BRPC and LAPC patients undergoing neoadjuvant FOLFIRINOX treatment and subsequent resection, 5-FU-based AT, negative RM, negative LN, preoperative BRPC status, better differentiation, and maintenance of standard-dose AT were associated with prolonged survival outcomes.

## Data availability statement

The raw data supporting the conclusions of this article will be made available by the authors, without undue reservation.

## Ethics statement

The studies involving human participants were reviewed and approved by Institutional Review Board of Seoul National University Hospital. Written informed consent for participation was not required for this study in accordance with the national legislation and the institutional requirements.

## Author contributions

Study concept and design: JC, MK, and WP. Acquisition or interpretation of data: JC, MK, NP, and JP. Draft of manuscript: JC, MK, and WP. Critical revision of the manuscript for important intellectual content: SL, IC, JR, Y-TK, WK, HK, and J-YJ. Study supervision: WP and SL. All authors contributed to the article and approved the submitted version.

## Acknowledgments

We appreciate the dedication of the pancreatic cancer multidisciplinary team consisting of Surgery, Radiation Oncology, Medical Oncology, Pathology, and Radiology.

## Conflict of interest

The authors declare that the research was conducted in the absence of any commercial or financial relationships that could be construed as a potential conflict of interest.

## Publisher’s note

All claims expressed in this article are solely those of the authors and do not necessarily represent those of their affiliated organizations, or those of the publisher, the editors and the reviewers. Any product that may be evaluated in this article, or claim that may be made by its manufacturer, is not guaranteed or endorsed by the publisher.
