# Supplementary material for: Proper adjuvant therapy in patients with borderline resectable and locally advanced pancreatic cancer who had received neoadjuvant FOLFIRINOX
Source: Front Oncol. 2022 Sep 20;12:945829. doi: 10.3389/fonc.2022.945829 (PMC9549517; doi:10.3389/fonc.2022.945829)
Supplement: Supplementary file 1 [file DataSheet_1.docx]

**Supplementary Table 1. Subgroup analysis of clinical outcomes according to neoadjuvant radiotherapy**

|  |  | **No neoadjuvant radiotherapy (n = 69)** | **Neoadjuvant radiotherapy**  **(n = 75)** | | |
| --- | --- | --- | --- | --- | --- |
|  |  |  | **Total** | | **P-value** |
| R0 resection | R0 | 59 (85.5%) | 66 (88%) | | 0.422 |
|  | R1 | 10 (14.5%) | 9 (12%) | |  |
| Death | HR (95% CI) | 1 | 0.786 (0.429–1.439) | | 0.435 |
| Recurrence | HR (95% CI) | 1 | 0.748 (0.437–1.281) | | 0.290 |
| Recurrence pattern | Locoregional | 15 (41.7%) | 4 (18.2%) | | 0.064 |
|  | Distant | 21 (58.3%) | 18 (81.8%) | |  |
|  |  |  |  |  |  |
|  |  |  | **5-FU based AT**  **(n = 32)** | **Non-5-FU based AT**  **(n = 43)** | **P-value** |
| R0 resection | R0 |  | 30 (93.8%) | 36 (83.7%) | 0.169 |
|  | R1 |  | 2 (6.2%) | 7 (16.3%) |  |
| Death | HR (95% CI) |  | 0.492 (0.188–1.290) | 1 | 0.149 |
| Recurrence | HR (95% CI) |  | 0.398 (0.167–0.948) | 1 | 0.035 |
| Recurrence pattern | Locoregional |  | 3 (33.3%) | 1 (7.7%) | 0.125 |
|  | Distant |  | 6 (66.7%) | 12 (92.3%) |  |

AT, adjuvant therapy; CI, confidence interval; FU, fluorouracil; HR, hazard ratio

**Supplementary Table 2. Subgroup analysis of prognostic factors for recurrence and death by multivariable Cox proportional hazards analysis except of patients who underwent adjuvant therapy without FOLFIRINOX among 5-FU based adjuvant therapy group**

|  | **Outcomes^*^** | | | |
| --- | --- | --- | --- | --- |
| **Covariates** | **Recurrence** | | **Death** | |
|  | **HR (95% CI)** | **p-value** | **HR (95% CI)** | **p-value** |
| **Adjuvant therapy with FOLFIRINOX (vs non 5-FU-based)^*^** | 0.29 (0.11–0.74) | 0.009 | 0.30 (0.01–0.92) | 0.035 |
| **Neoadjuvant chemotherapy dose reduction (vs standard dose)** | 1.23 (0.53–2.86) | 0.637 | 0.94 (0.34–2.78) | 0.961 |
| **LA as preoperative resectability (vs Resectable or BR)** | 2.35 (0.75–7.37) | 0.144 | 1.92 (0.48–7.72) | 0.358 |
| **Objective response to neoadjuvant therapy (vs stable disease)** | 1.55 (0.47–5.14) | 0.474 | 0.90 (0.21–3.80) | 0.887 |
| **Extended resection (vs standard resection)** | 1.24 (0.52–2.96) | 0.628 | 1.99 (0.63–6.29) | 0.241 |
| **CAP score 0–2 (vs 3)** | 0.75 (0.29–1.95) | 0.550 | 1.43 (0.47–4.55) | 0.550 |
| **Resection margin R1 (vs R0)** | 5.89 (2.01–17.21) | 0.001 | 5.20 (1.64–16.46) | 0.005 |
| **Stage ypT2–4 (vs ypT0 or ypT1)** | 1.49 (0.63–3.56) | 0.368 | 1.58 (0.53–4.73) | 0.414 |
| **Stage ypN1 or ypN2 (vs ypN0)** | 2.11 (0.77–5.79) | 0.147 | 2.95 (0.92–9.45) | 0.068 |
| **Postoperative CA19-9 normalization (vs not)** | 0.433 (0.19–1.01) | 0.053 | 0.334 (0.18–0.95) | 0.040 |
| **Differentiation PD (vs WD~MD)** | 3.05 (0.87–10.72) | 0.083 | 15.22 (3.20–72.39) | 0.001 |
| **Adjuvant chemotherapy dose reduction (vs standard dose)** | 2.56 (0.46–14.35) | 0.286 | 5.52 (0.96–31.65) | 0.055 |
| **Performance before adjuvant therapy; ECOG 0 or 1 (vs ECOG ≥ 2)** | 4.11 (0.85–19.77) | 0.078 | 2.57 (0.59–11.11) | 0.207 |

BR, borderline resectable; CAP, College of American Pathologists; ECOG, Eastern Cooperative Oncology Group; FU, fluorouracil; LA, locally advanced; MD, moderately differentiated; PD, poorly differentiated; RT, radiotherapy; WD, well differentiated

^*^Comparison of non 5-FU-based adjuvant therapy group with the subgroup of patients who underwent adjuvant therapy with FOLFIRINOX among 5-FU based adjuvant therapy group.
